# Naringenin ameliorates collagen‐induced arthritis through activating AMPK‐mediated autophagy in macrophages

**DOI:** 10.1002/iid3.983

**Published:** 2023-10-20

**Authors:** Wei Zhang, Yuan Zhang, Jianguang Zhang, Chunbiao Deng, Chao Zhang

**Affiliations:** ^1^ Department of Orthopedic Affiliated Hospital of North Sichuan Medical College Nanchong China

**Keywords:** AMPK, autophagy, macrophage, Naringenin, rheumatoid arthritis

## Abstract

**Background:**

Naringenin is widely recognized for its notable attributes, including anti‐inflammatory, anti‐cancer, and immunomodulatory activities. However, its specific implications for rheumatoid arthritis (RA) and the underlying mechanisms remain to be explored. This study aimed to investigate the therapeutic efficacy and pharmacological mechanism of Naringenin in the treatment of collagen‐induced arthritis (CIA).

**Methods:**

A CIA model was established in DBA/1 mice, and various doses of Naringenin were administered orally to assess its impact on RA. The study also involved lipopolysaccharides (LPS)‐induced RAW264.7 cells to further evaluate the effects of Naringenin. Mechanistic studies were conducted to elucidate the signaling pathways involved in Naringenin's actions.

**Results:**

Naringenin significantly alleviated foot inflammation in DBA/1 CIA mice and attenuated the levels of pro‐inflammatory cytokines in serum. It also enhanced antioxidant capacity in the CIA model. In vitro studies with LPS‐induced RAW264.7 cells demonstrated that Naringenin attenuated pro‐inflammatory cytokines and reactive oxygen species (ROS) levels. Mechanistic studies confirmed that Naringenin activated autophagy and increased autophagic flux. Blocking autophagy, either by silencing Atg5 or inhibiting autophagolysosome using chloroquine, effectively counteracted the impact of Naringenin on pro‐inflammatory cytokines. Further exploration revealed that Naringenin activated the AMPK/ULK1 signaling pathway, and inhibition of AMPK reversed the initiation of autophagy and reduced pro‐inflammatory cytokine secretion induced by Naringenin.

**Conclusions:**

This study unveils a novel mechanism by which Naringenin may be used to treat RA. It demonstrates the therapeutic efficacy of Naringenin in a CIA model by reducing inflammation, modulating cytokine levels, and enhancing antioxidant capacity. Moreover, the activation of autophagy through the AMPK/ULK1 signaling pathway appears to play a critical role in Naringenin's anti‐inflammatory effects. These findings suggest potential strategies for the development of anti‐rheumatic medications based on Naringenin.

## Graphical abstract

Naringenin ameliorates arthritis through activating AMPK/ULK1 signaling pathway. Inhibition of AMPK similarly reverses initiation of autophagy and reducution pro‐inflammatory cytokines secretion by Naringenin.

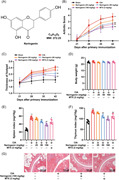

## 1 INTRODUCTION

Rheumatoid arthritis (RA) is the most common autoimmune and degenerative joint disease associated with immune hyperactivation and synovitis.^1^, ^2^ The pharmacological agents commonly employed in the treatment of RA encompass NSAIDs, disease‐modifying antirheumatic drugs (DMARDs), and glucocorticoids. Common DMARDs, such as tumor necrosis factor (TNF) inhibitors and interleukin‐6 (IL‐6) receptor inhibitors, are currently used worldwide for the treatment of RA, but do not control disease progression in all patients.^3^, ^4^, ^5^ In addition, most of these drugs have side effects such as infection and renal toxicity during long‐term use.^2^, ^6^ Hence, there is a pressing requirement for the development of novel pharmaceuticals that exhibit reduced adverse effects and enhanced efficacy in treating RA.

Pro‐inflammatory factors play an important role in the progression of RA.^7^, ^8^ Excessive TNF‐α enhances inflammatory response, activates synovial fibroblasts, leads to synovial hyperplasia, and damages articular cartilage.^9^ IL‐1β induces the proliferation of synovial fibroblasts, which is closely related to joint synovial injury, and enhances the expression of cyclooxygenase‐2 (COX‐2) in synovial fibroblasts.^10^ High levels of transforming growth factor‐β (TGF‐β) in serum enhance osteoclast bone resorption, mediates synovial cell proliferation, and endothelial angiogenesis.^11^, ^12^ In addition, studies have shown that IL‐17 may be a determinant of RA pathogenesis.^13^, ^14^ Ultimately, these pro‐inflammatory cytokines interact and promote each other to form a strong inflammatory cascade that induces synovial hyperplasia and enhances histopathological changes in RA.^7^, ^15^ The pro‐inflammatory factors associated with RA are secreted mainly by activated macrophages present in the articular tissues of RA patients.^16^ In addition, the activation of macrophages can also activate osteoclasts, causing pathological absorption and destruction of bone and cartilage.^17^ Therefore, inhibition of macrophage activation may be a potential new strategy for RA treatment.

Reactive oxygen species (ROS) maintain the redox state of cells and play an important role in the process of signal transduction, growth, proliferation, apoptosis, and differentiation of macrophages.^18^, ^19^, ^20^ The occurrence of RA induces oxidative stress, which contributes to the activation and maintenance of macrophages.^21^, ^22^ Therefore, inhibition of oxidative stress can effectively control the activation of macrophages.^23^ The initiation of autophagy plays an important role in maintaining redox balance.^24^ Autophagy is a physiological process in eukaryotic systems that plays an important role in adaptation to oxidative stress by degrading damaged organelles and oxidative damaged macromolecules.^25^, ^26^, ^27^ In oxidative stress response, autophagy can eliminate the oxidative components of cells by removing damaged proteins or organelles to reduce the accumulation of oxidative stress.^28^ Thus, triggering autophagy helps maintain the redox balance and thus restricts macrophage activation.

Naringenin (4, 5, 7‐trihydroxyflavone) is a flavonoid compound found in plants and abundant in citrus fruits.^29^, ^30^ It was confirmed that Naringenin has a variety of pharmacological activities, such as anti‐inflammatory, antioxidant, antitumor, immune regulation, and so forth.^31^, ^32^, ^33^, ^34^ While previous studies have reported the anti‐RA effects of Naringenin and Naringin, these studies have focused mainly on their in vivo effects and modulation of dendritic cell function, lacking a comprehensive exploration of the anti‐inflammatory mechanism of Naringenin.^35^, ^36^, ^37^ This study employed collagen‐induced arthritis (CIA) mouse model to examine the in vivo therapeutic potential of Naringenin. Furthermore, we assessed the ability of Naringenin to enhance autophagy in lipopolysaccharides (LPS)‐induced RAW264.7 cells and elucidated the associated molecular mechanisms.

## 2 MATERIALS AND METHODS

### 2.1 Reagents and antibodies

We procured Naringenin (purity: 99%) and LPS (purity: 99%) from Solarbio Science & Technology with product codes #S90147 and #L8880, respectively. MedChemExpress LLC provided CQ (HY‐17589A) and Compound C (HY‐13418A). ABclonal Biotechnology Co. Ltd. provided primary antibodies against acetyl‐CoA carboxylase (ACC, A15606), Unc‐51‐like kinase 1 (ULK1, A8529), and β‐actin (AC026) and secondary antibodies horseradish peroxidase (HRP) goat anti‐mouse IgG (H + L) and HRP goat anti‐rabbit IgG (H + L). Cell Signaling Technology provided primary antibodies against AMP^−^activated protein kinase α (AMPKα, #2532), phosphorylated AMPKα at Thr172 (#2535), phosphorylated ACC at Ser79 (#11818), phosphorylated ULK1 at Ser555 (#5869), and microtubule‐associated protein 1A/1B‐light chain 3A/B (LC3A/B, #4108). Abcam provided the antibody against p62/sqstm1 (AB56416).

### 2.2 Animals

We obtained male DBA/1 mice, free from pathogens, from Beijing Weitong Lihua Experimental Animal Co. Ltd. (SCXK2012‐0001). These mice were housed in a controlled environment with a 12‐h light/dark cycle, within a laminar flow cabinet. They were provided with specific pathogen‐free (SPF) laboratory chow and water ad libitum. All experimental procedures were conducted following the guidelines established by The Animal Ethics Committee of Affiliated Hospital of North Sichuan Medical College.

### 2.3 Induction of CIA

Preparation of emulsion containing type II collagen (CII): Chicken CII was dissolved in 0.1 µM acetic acid at a concentration of 2 mg/mL and left overnight at 4°C. Subsequently, it was combined with complete Freund's adjuvant (C9301; Sigma). Male DBA/1 mice, aged 6–8 weeks, were subjected to immunization with 100 µg of chicken CII (100 µL). The initiation of the first immunization was designated as Day 0. On Day 21, the mice were administered a booster containing an equal amount of chicken CII emulsified in Freund's incomplete adjuvant.^38^, ^39^ The experimental design involved the categorization of mice with CIA into six distinct groups (*n* = 10 per group): a sham group, a model group, and three groups treated with varying doses of Naringenin (25, 50, 100 mg/kg/day), and a methotrexate (MTX) group (2 mg/kg/every 3 days) serving as the positive control. To induce anesthesia, a 2% isoflurane inhalation anesthetic was employed. Starting from Day 26 to Day 46, three doses of Naringenin and MTX, both dissolved in corn oil or the respective vehicle (corn oil) were orally administered via gavage to the mice with CIA. Likewise, the sham group received vehicle (corn oil) from Day 26 to Day 46. On Day 46, blood samples of 0.5 mL were taken from each animal through cardiac puncture with anesthesia application.

Starting from Day 21 following the initial immunization, we assessed the severity of arthritis on a weekly basis using a scoring system ranging from 0 to 4 for each limb: 0 indicating normal condition, 1 representing definite redness and swelling in the ankle or one digit, 2 denoting involvement of two joints, 3 indicating involvement of more than two joints, and 4 representing severe arthritis affecting the entire paw and all digits. Furthermore, we measured the thickness of the footpads each week. In cases where the mice experienced a weight loss exceeding 20% or developed infected and purulent paw and digits, we conducted euthanasia to minimize suffering. On the 46th day, mice were humanely euthanized using CO_2_ asphyxiation. Subsequently, the spleens and thymus were extracted and weighed to assess the occurrence of splenomegaly and thymus enlargement. To determine the spleen and thymus index, which represents the ratio of spleen or thymus weight to body weight multiplied by 10, the following calculation was performed.

### 2.4 Histopathologic examination

After the mice were killed, the joint or paw tissues were removed, fixed in 10% formalin, decalcified in a 5% EDTA‐2Na solution, embedded in paraffin, sectioned, and then stained with hematoxilin and eosin (H&E) for light microscopy observation.

### 2.5 Measurement of cytokine

The blood samples were allowed to coagulate naturally at ambient temperature, after which they were subjected to centrifugation to separate the serum. The levels of TNF‐α, IL‐1β, IL‐17, and TGF‐β in the serum samples were determined using a Mouse TNF‐α, IL‐1β, IL‐17, and TGF‐β ELISA kit (ABclonal), following the instructions provided by the manufacturer. The concentrations of cytokines (pg/mL) were calculated based on the optical density (OD) measured at 450 nm.

### 2.6 Measurement of superoxide dismutase (SOD) activity, malonaldehyde (MDA), and glutathione (GSH)

To evaluate the total SOD activities, we employed a commercially available Total Superoxide Dismutase Assay Kit (Beyotime, S0109). The determination of total GSH levels was conducted using a GSH detection kit (Beyotime, S0052). To assess the extent of lipid peroxidation, the MDA content was quantified utilizing a lipid peroxidation MDA assay kit (Beyotime, S0131S). The experimental procedures for these assays strictly followed the manufacturer's instructions.

### 2.7 Cell culture

The RAW264.7 cell line was acquired from the American Type Culture Collection (ATCC) and maintained in a humidified incubator at 37°C with 5% CO_2_. The cells were cultured in RPMI Medium 1640 supplemented with 10% fetal bovine serum (FBS), 100 U/mL of penicillin, and 100 μg/mL of streptomycin. The in vitro experimental design consisted of different groups: control group, group treated with LPS (1 μg/mL), groups treated with different concentrations of naringenin (12.5, 25, 50, and 100 μM), groups treated with CQ (50 μM), or Compound C (10 μM).

### 2.8 Cell viability

We assessed the cytotoxic effects of Naringenin on RAW264.7 cells using the 3‐(4, 5‐dimethylthiazolyl‐2)‐2,5‐diphenyltetrazolium bromide (MTT) assay. Each concentration of Naringenin was tested in triplicate experiments conducted in parallel. Control cells were treated with culture media containing the appropriate solvent control. Following a 24‐h incubation period, 20 μL of a 5 mg/mL MTT solution was added to each well, and the cells were further incubated at 37°C for 4 h. Subsequently, the supernatant was carefully removed, and 150 μL of dimethyl sulfoxide (DMSO) was added to each well to dissolve the purple formazan crystals. The absorbance was then measured at 570 nm using an ELx800 automated microplate reader (BioTek Instruments, Inc.). These experimental procedures were repeated three times for each concentration of Naringenin used.

### 2.9 RNA isolation and real‐time quantitative polymerase‐chain‐reaction (qPCR) analysis

Total RNA extraction was conducted from tissues or cells utilizing the Trizol Reagent, following the manufacturer's instructions. Subsequently, mRNA was reverse‐transcribed into cDNA using the 5× All‐In‐One RT MasterMix. The quantification of gene expression was performed using the EvaGreen qPCR MasterMix on a MiniOpticon Real‐Time PCR Detection System (BioRad Laboratories). The primer sequences employed are provided below.

For Mouse TNF‐α:

Forward primer: 5′‐ATGAGCACAGAAAGCATGATCCGC‐3′

Reverse primer: 5′‐AAAGTAGACCTGCCCGGACTC‐3′

For Mouse IL‐1β:

Forward primer: 5′‐CCAAGCTTCCTTGTGCAAGTA‐3′

Reverse primer: 5′‐AAGCCCAAAGTCCATCAGTGG‐3′

For Mouse TGF‐β1:

Forward primer: 5′‐CTCCCGTGGCTTCTAGTGC‐3′

Reverse primer: 5′‐GCCTTAGTTTGGACAGGATCTG‐3′

For Mouse GAPDH:

Forward primer: 5′‐GGTGAAGGTCGGTGTGAACG‐3′

Reverse primer: 5′‐CTCGCTCCTGGAAGATGGTG‐3′

### 2.10 Measurement of ROS formation

We employed the fluorescent dye 2,7‐dichlorofluorescein diacetate (DCFH‐DA, sourced from Beyotime Institute of Biotechnology) to quantify the levels of ROS. The RAW 264.7 cells were harvested and subjected to a 30‐min incubation with DCFH‐DA at 37°C, in the absence of light. Flow cytometry was utilized to measure the fluorescence intensity.

### 2.11 Western blot

We employed western blot analysis to evaluate the protein expression levels in RAW264.7 cells. Specifically, we examined the levels of p62, LC3A/B, p‐AMPK (Thr 172), AMPK, p‐ACC (Ser 79), ACC, p‐ULK1 (Ser 555), and ULK1. Following the preparation of whole cell lysates, we performed protein separation using a 10% sodium dodecyl sulfate‐polyacrylamide gel electrophoresis (SDS‐PAGE) system operated at 100 V. The proteins were subsequently transferred onto nitrocellulose membranes via a transfer apparatus. We then proceeded to wash the membranes with PBST and block them at room temperature for 60 min using 5% nonfat dry milk in PBST. For antibody incubation, we employed primary antibodies targeting p62, LC3A/B, p‐AMPK (Thr 172), AMPK, p‐ACC (Ser 79), ACC, p‐ULK1 (Ser 555), ULK1, and β‐actin. These antibodies were incubated overnight at 4°C on a platform shaker. Following the incubation, we washed the membranes and applied the appropriate HRP^−^conjugated secondary antibody. Finally, we used an enhanced chemiluminescence (ECL) substrate solution (Thermo Scientific) and visualized the protein bands using a Fully Automatic Chemiluminescence Image Analysis System (Tanon).

### 2.12 Plasmids

To examine the impact of Naringenin on autophagic flux, the GFP‐RFP‐LC3 plasmid (Addgene) was employed. To elucidate the involvement of autophagy and AMPK signaling in the suppression of macrophage activation by Naringenin, we generated plasmids containing targeted shRNA sequences targeting Atg5 and Ampkα, respectively. The lentivirus‐based pLKO vector was utilized to express the shRNA. The specific shRNA sequences employed in this study were obtained from the Sigma shRNA Mission library and are listed below:

shAtg5 (5′‐CCGGAGCCTCCTCTTCTCGTGAAATCTCGAGATTTCACGAGAAGAGGAGGCTTTTTTG‐3′)

shAmpkα (5′‐CCGGTTGTTGGATTTCCGTAGTATTCTCGAGAATACTACGGAAATCCAACAATTTTTG‐3′)

Sequences of all oligonucleotides used in this study are available upon request.

### 2.13 Transient transfection and lentivirus infection

Cells were cultured in 6‐well plates before transfection. Once the cells attained an approximate confluence of 80%, transfection was carried out utilizing Lipofectamine 2000 (Invitrogen) for transient transfection. To generate lentivirus, HEK293T cells were transfected with pLKO plasmids, along with pMD2G and psPAX2 plasmids. After 48 h of transfection, the viral supernatant was collected and used to infect target cells. Puromycin was employed for selection purposes.

### 2.14 Statistical analysis

The study collected data through a minimum of three separate experiments, and all findings presented are expressed as the mean ± standard deviation (SD). The statistical comparison between groups was conducted using a one‐way analysis of variance (ANOVA) test. Specific information regarding each statistical analysis performed can be found in the figure captions. Statistical significance was defined as differences with *p* values below .05.

## 3 RESULTS

### 3.1 Naringenin exhibited beneficial effects in alleviating CIA in DBA/1 mice

**Figure 1:**
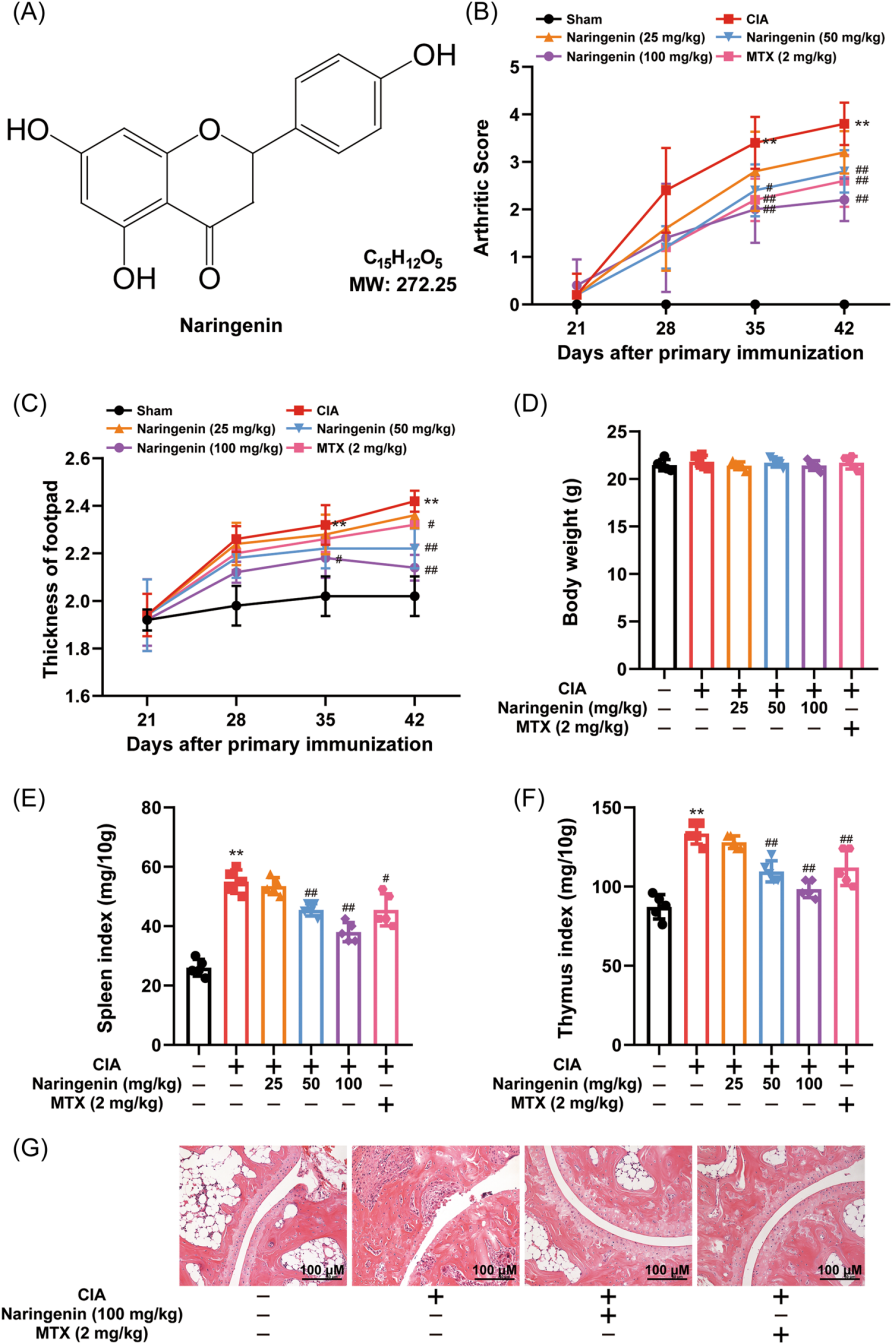
Naringenin ameliorated CIA in DBA/1 mice. (A) Chemical structure of Naringenin. CIA mice were administered vehicle, Naringenin (25, 50, 100 mg/kg) or MTX (2 mg/kg every 3 days) on Day 26. (B and C) The arthritis score and footpad thickness were measured every week after secondary immunization (*n* = 5). (D) Body weights of each group on Day 46 (*n* = 5). (E and F) The spleen and thymus index (ratio of spleen and thymus weight to body weight × 10) was calculated after the mice were killed (*n* = 5). (G) Representative photographs of HE‐stained sections of ankle joints. Scale bar = 100 μm (×200). Data are presented as mean ± SD. Differences were compared by the paired *t* test. **p < .01 compared with normal mice; ^#^ *p* < .05, ^##^ *p* < .01 compared with CIA mice. CIA, collagen‐induced arthritis; HE, hematoxilin and eosin; MTX, methotrexate; SD, standard deviation.

From Day 26 to 46, both Naringenin and MTX were administered to the mice with CIA, and the efficacy of Naringenin was evaluated by weekly measurements of clinical arthritis scores and footpad thickness. The results exhibited a significant reduction in clinical arthritis scores and footpad thickness upon Naringenin treatment (Figure 1B,C), while no notable effect on body weight was observed (Figure 1D). Following sacrifice, the spleen and thymus indices were measured to assess the impact of Naringenin on splenomegaly and thymus enlargement induced by immune abnormalities in CIA mice (Figure 1E,F). Histological analysis of H&E‐stained sections revealed severe joint damage characterized by infiltration of inflammatory cells in the ankle joints of CIA mice. Remarkably, Naringenin treatment significantly ameliorated inflammation and attenuated joint destruction (Figure 1G).

### 3.2 Naringenin suppressed the production of pro‐inflammatory mediators in the serum of mice with CIA

**Figure 2:**
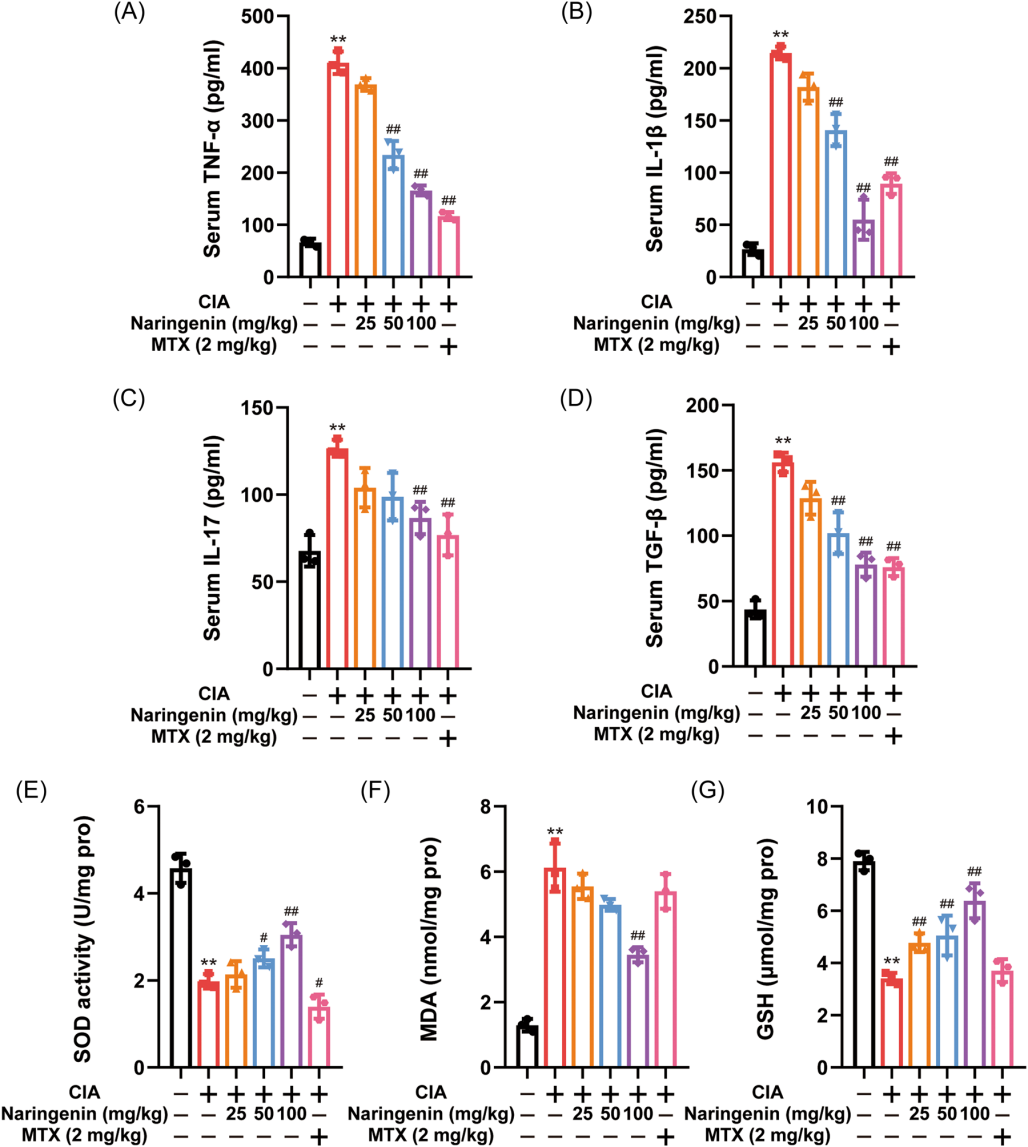
Naringenin reduced pro‐inflammatory cytokines secretion and oxidative stress in CIA mice. (A–D) The TNF‐α, IL‐1β, IL‐17, and TGF‐β levels in serum were measured by ELISA (*n* = 3). (E–G) The SOD activity, MDA, and GSH levels were measured in serum (*n* = 3). Data are presented as mean ± SD. Differences were compared by the paired *t* test. **p < .01 compared with normal mice; ^#^ *p* < .05, ^##^ *p* < .01 compared with CIA mice. CIA, collagen‐induced arthritis; ELISA, enzyme‐linked immunosorbent assay; GSH, glutathione; IL, interleukin; MDA, malonaldehyde; MTX, methotrexate; SD, standard deviation; SOD, superoxide dismutase; TGF‐β, transforming growth factor‐β; TNF‐α, tumor necrosis factor‐α.

The serum levels of pro‐inflammatory factors (TNF‐α, IL‐1β, IL‐17, and TGF‐β) in CIA mice were determined by ELISA to elucidate the mechanism of Naringenin in alleviating CIA. The findings demonstrated that CIA mice exhibited elevated levels of pro‐inflammatory factors in their serum when compared to normal mice. Notably, the Naringenin treatment group (at doses of 100 mg/kg) and the MTX group (at a dose of 2 mg/kg) showed significant reductions in the secretion levels of TNF‐α, IL‐1β, IL‐17, and TGF‐β compared to the CIA mice (Figure 2A–D). Moreover, to elucidate the antioxidative effect of Naringenin on CIA mice, the activities of SOD and GSH, as well as the concentration of MDA, were assessed. It was observed that CIA mice displayed an increased concentration of MDA, whereas the activity of SOD and GSH decreased in comparison to normal mice (Figure 2E–G). However, this effect was reversed after 100 mg/kg of Naringenin treatment.

### 3.3 Naringenin mitigated the expression of pro‐inflammatory mediators and ROS in RAW264.7 cells stimulated with LPS

**Figure 3:**
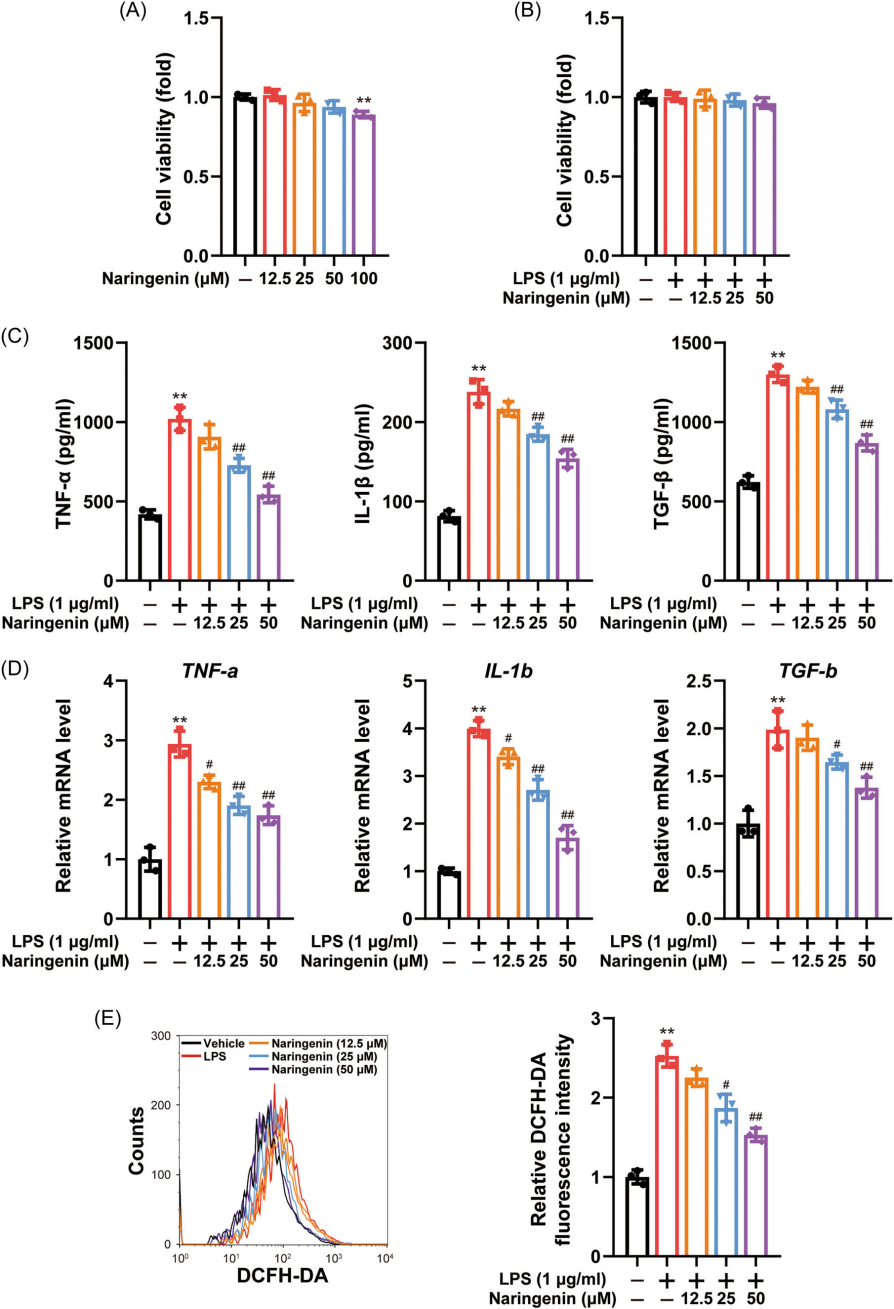
Naringenin inhibited pro‐inflammatory cytokines and ROS production in LPS‐induced RAW264.7 cells. (A and B) The cytotoxicity of Naringenin on RAW264.7 cells was detected with or without LPS (*n* = 3). (C) The TNF‐α, IL‐1β, and TGF‐β levels in culture supernatants were measured by ELISA (*n* = 3). (D) The mRNA levels of *TNF‐a*, *IL‐1b*, and *TGF‐b* were measured by real‐time qPCR (*n* = 3). (E) The RAW 264.7 cells were loaded with DCFH/DA. The geometric mean DCF fluorescence was measured by flow cytometry (*n* = 3). Data are presented as mean ± SD. Differences were compared by the paired *t* test. **p < .01 compared with control group; ^#^ *p* < .05, ^##^ *p* < .01 compared with LPS‐treated group. DCFH/DA, 2,7‐dichlorofluorescein diacetate; ELISA, enzyme‐linked immunosorbent assay; GSH, glutathione; IL, interleukin; LPS, lipopolysaccharide; MDA, malonaldehyde; MTX, methotrexate; qPCR, real‐time quantitative polymerase‐chain‐reaction; ROS, reactive oxygen species; SD, standard deviation; SOD, superoxide dismutase; TGF‐β, transforming growth factor‐β; TNF‐α, tumor necrosis factor‐α.

To assess the impact of Naringenin on RAW264.7 cell viability, we employed the MTT assay. Remarkably, no discernible impact on cell viability was observed when treating with 50 μM Naringenin (Figure 3A,B). However, the cell viability displayed a decline trend as the concentration of Naringenin increased. Consequently, for subsequent in vitro experiments, we employed Naringenin concentrations of 12.5, 25, and 50 μM in RAW264.7 cells. We investigated the effects of Naringenin on the secretion of TNF‐α, IL‐1β, and TGF‐β in LPS‐induced RAW264.7 cells. The outcomes revealed that LPS treatment augmented the secretion of TNF‐α, IL‐1β, and TGF‐β, whereas 25 and 50 μM Naringenin significantly attenuated the secretion of these cytokines in LPS‐induced RAW264.7 cells (Figure 3C). Correspondingly, the mRNA levels of TNF‐α, IL‐1β, and TGF‐β were significantly suppressed by Naringenin (Figure 3D). Furthermore, we assessed the levels of ROS, which revealed that LPS treatment elevated ROS levels, whereas 25 and 50 μM Naringenin significantly mitigated ROS levels in LPS‐induced RAW264.7 cells (Figure 3E). Collectively, these findings indicate that Naringenin inhibits macrophage activation and oxidative stress induced by LPS.

### 3.4 Naringenin reduced the level of pro‐inflammatory factors via triggering autophagy in LPS‐induced RAW264.7 cells

**Figure 4:**
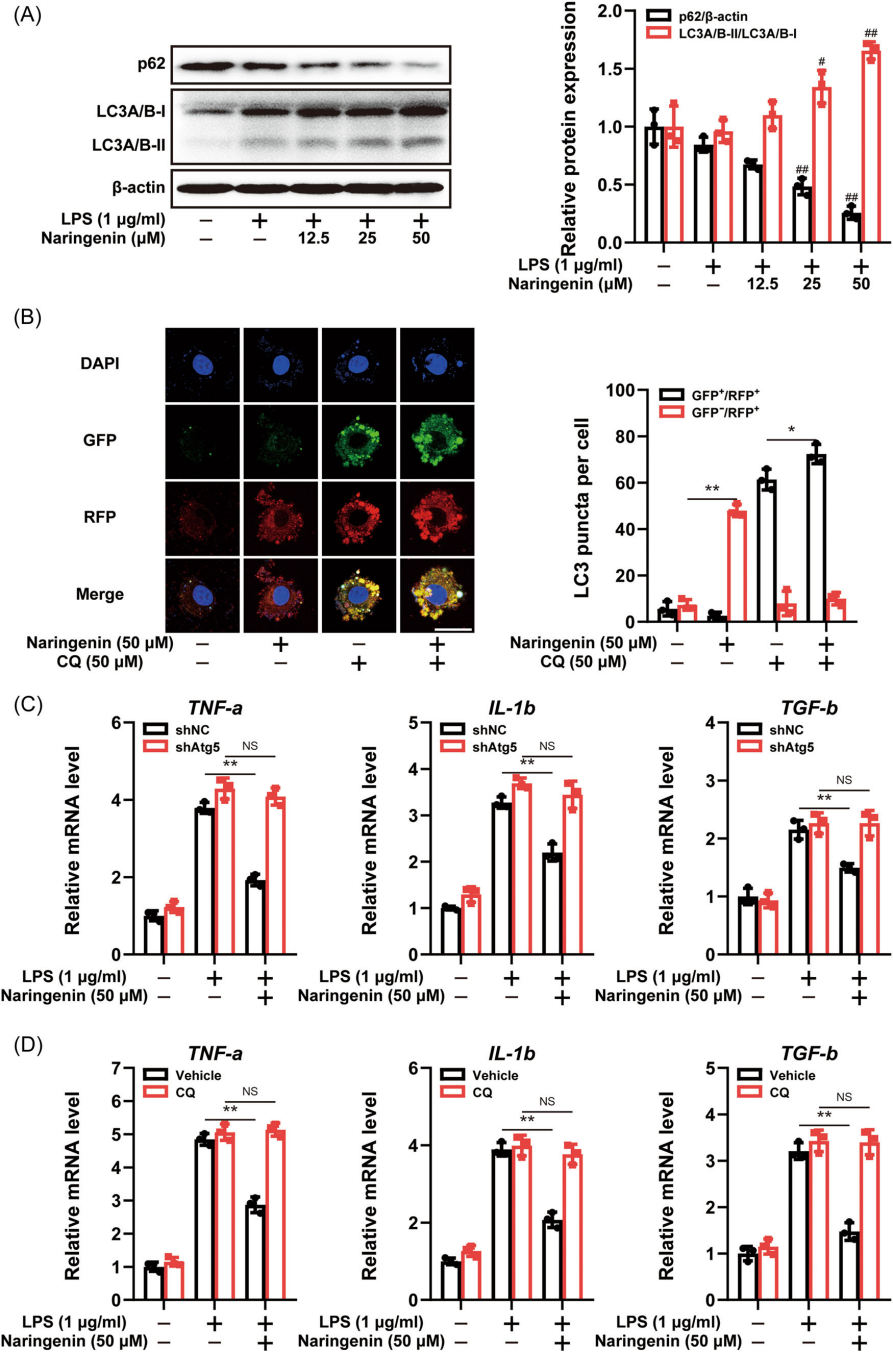
Naringenin triggered autophagy to reduce pro‐inflammatory cytokines production in LPS‐induced RAW264.7 cells. (A) Immunoblot analysis of p62 and LC3A/B expression in LPS‐induced RAW264.7 cells. Densitometric analysis was performed to determine the relative ratios of each protein. Data are presented as mean ± SD (*n* = 3). Differences were compared by the paired *t* test. ^#^ *p* < .05, ^##^ *p* < .01 compared with LPS‐treated group. (B) RAW264.7 cells were transfected with GFP‐RFP‐LC3 for 24 h, after transfection cells were treated with Naringenin (50 μM) alone or with CQ (50 μM). Cells were observed under a confocal microscopy. Representative images are shown. Scale bars, 10 µm (×1000). Data are presented as mean ± SD (*n* = 3). Differences were compared by the paired *t* test. *p < .05, **p < .01. (C) The mRNA levels of *TNF‐a*, *IL‐1b*, and *TGF‐b* were measured by real‐time qPCR in LPS‐induced RAW264.7 cells after knockdown of Atg5. Data are presented as mean ± SD (*n* = 3). Differences were compared by the paired *t* test.**p < .01. (D) The mRNA levels of *TNF‐a*, *IL‐1b*, and *TGF‐b* were measured by real‐time qPCR after treatment of CQ. Data are presented as mean ± SD (*n* = 3). Differences were compared by the paired *t* test. **p < .01. IL, interleukin; LPS, lipopolysaccharide; qPCR, real‐time quantitative polymerase‐chain‐reaction; SD, standard deviation; TGF‐β, transforming growth factor‐β; TNF‐α, tumor necrosis factor‐α.

Autophagy plays a crucial role in preserving cellular redox equilibrium and modulating the inflammatory response. To elucidate the underlying mechanism through which Naringenin inhibits macrophage activation, we conducted an investigation into the impact of Naringenin on autophagy. We found that after Naringenin treatment, the level of LC3A/B‐II protein increased gradually (Figure 4A). These results suggest that Naringenin can increase the autophagosome formation. To determine whether Naringenin treatment increases autophagy flux, degradation of p62 was studied. As shown in Figure 4A, p62 protein levels were significantly reduced during Naringenin treatment. To evaluate the autophagic flux status, we employed the GFP‐RFP‐LC3 plasmid. Upon treatment with Naringenin, there was an observed increase in the number of GFP^‐^RFP^+^ (red) puncta, indicating enhanced autolysosome formation. Conversely, in the group treated with CQ, an inhibitor of autophagolysosomes, a significant number of GFP^+^RFP^+^ (yellow) puncta and a few GFP^‐^RFP^+^ (red) puncta were observed, suggesting blockade of autophagolysosome formation. Notably, the combination of Naringenin and CQ further intensified the presence of GFP^+^RFP^+^ (yellow) puncta (Figure 4B). These results implies that Naringenin augments autophagic flux.

To investigate whether Naringenin inhibits macrophage activation by inducing autophagy, we either knocked down Atg5, a pivotal gene involved in autophagosome formation, or added CQ to hinder autophagolysosome formation. The findings demonstrated that Atg5 knockdown or CQ treatment, in conjunction with Naringenin, reversed the downregulation of TNF‐α, IL‐1β, and TGF‐β mRNA levels caused by Naringenin. However, Atg5 knockdown or CQ treatment alone did not exhibit any significant impact on the expression of pro‐inflammatory factors mRNA (Figure 4C,D). These results suggest that Naringenin inhibits LPS‐induced macrophage activation through the initiation of autophagy.

### 3.5 Naringenin exhibited anti‐inflammatory effects and induced autophagy by activating the AMPK signaling pathway

**Figure 5:**
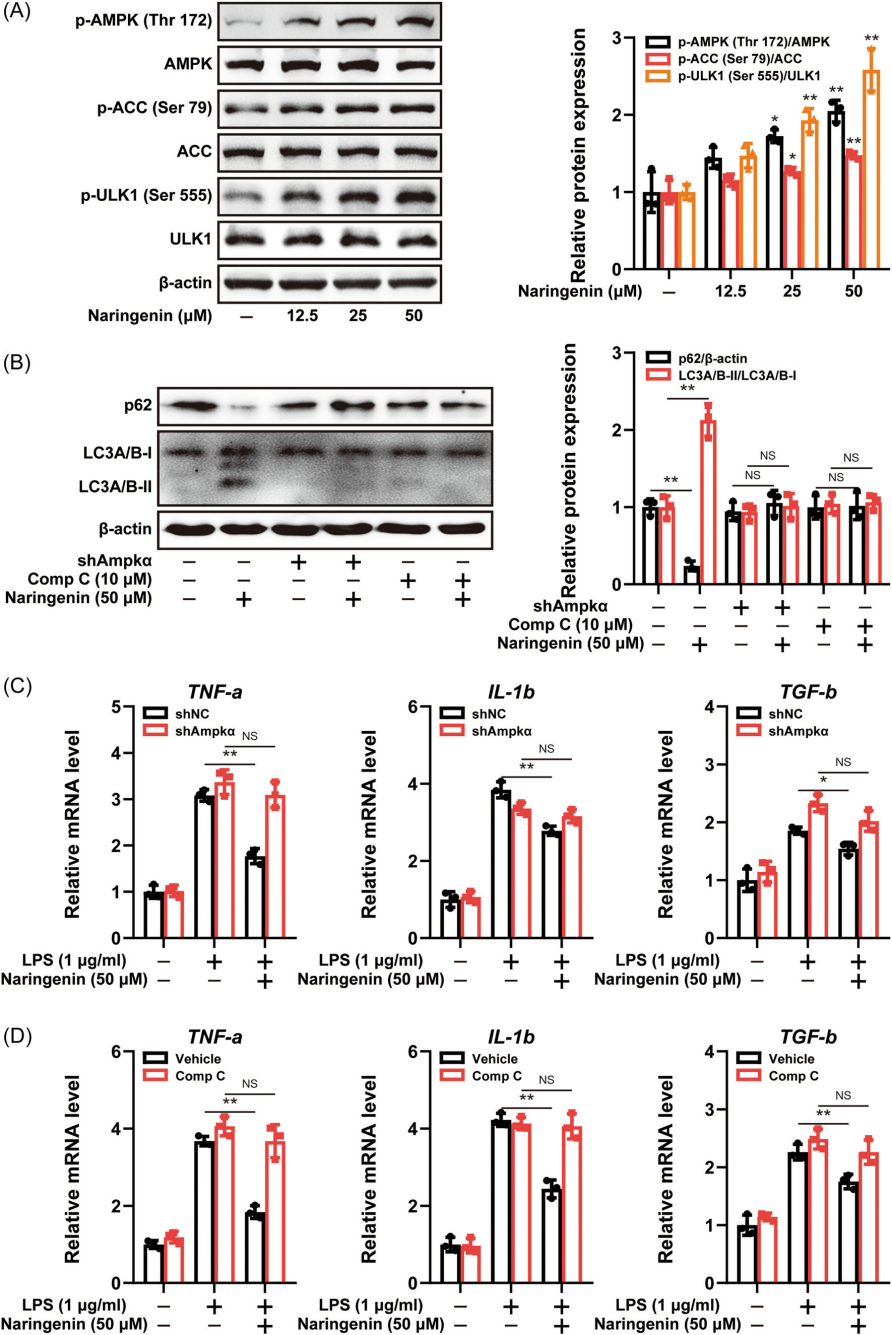
Naringenin activated AMPK signaling to trigger autophagy and reduce pro‐inflammatory cytokines production in LPS‐induced RAW264.7 cells. (A) Immunoblot analysis of p‐AMPK (Thr 172), AMPK, p‐ACC (Ser 79), ACC, p‐ULK1 (Ser 555), and ULK1 expression in LPS‐induced RAW264.7 cells. Densitometric analysis was performed to determine the relative ratios of each protein. Data are presented as mean ± SD (*n* = 3). Differences were compared by the paired *t* test. *p < .05, **p < .01 compared with control group. (B) Immunoblot analysis of p62 and LC3A/B expression in LPS‐induced RAW264.7 cells after knockdown of Ampkα or treatment of Compound C. Densitometric analysis was performed to determine the relative ratios of each protein. Data are presented as mean ± SD (*n* = 3). Differences were compared by the paired *t* test.**p < .01. (C) The mRNA levels of *TNF‐a*, *IL‐1b*, and *TGF‐b* were measured by real‐time qPCR in LPS‐induced RAW264.7 cells after knockdown of Ampkα. Data are presented as mean ± SD (*n* = 3). Differences were compared by the paired *t* test. *p < .05, **p < .01. (D) The mRNA levels of *TNF‐a*, *IL‐1b*, and *TGF‐b* were measured by real‐time qPCR after treatment of Compound C. Data are presented as mean ± SD (*n* = 3). Differences were compared by the paired *t* test. **p < .01. IL, interleukin; LPS, lipopolysaccharide; qPCR, real‐time quantitative polymerase‐chain‐reaction; SD, standard deviation; TGF‐β, transforming growth factor‐β; TNF‐α, tumor necrosis factor‐α.

Prior research has indicated that the AMPK/ULK‐1 signaling pathway plays a pivotal role in the regulation of autophagy, which is closely associated with RA. In this study, we aimed to elucidate the mechanism underlying Naringenin‐induced autophagy by evaluating the phosphorylation levels of AMPK, ACC, and ULK1 proteins. As depicted in Figure 5A, Naringenin upregulated the expression of p‐AMPK (Thr 172), p‐ACC (Ser 79), and p‐ULK1 (Ser 555), thus suggesting the activation of the AMPK signaling pathway. To further investigate whether Naringenin initiates autophagy via AMPK activation, we examined the impact of Naringenin on LC3A/B and p62 protein expression following Ampkα knockdown or treatment with the AMPK inhibitor Compound C. The results showed that the Ampkα knockdown or Compound C individually did not yield any impact on the expression of LC3A/B‐II and p62 proteins. However, the combination of Ampkα knockdown or Compound C with Naringenin exhibited a reversal effect on the upregulation of LC3A/B‐II and downregulation of p62 protein expression induced by Naringenin (Figure 5B). Furthermore, it was observed that the mRNA levels of TNF‐α, IL‐1β, and TGF‐β, which were decreased by Naringenin, were restored by the treatment of Ampkα knockdown or Compound C. Conversely, Ampkα knockdown or treatment with CQ alone did not exert a significant influence on the mRNA expression of pro‐inflammatory factors (Figure 5C,D). These results indicate that Naringenin initiates autophagy and inhibits LPS‐induced macrophage activation by activating the AMPK signaling pathway.

## 4 DISCUSSION

Naringenin is a flavonoid compound widely found in plants with antioxidant, anti‐inflammatory, and immunomodulatory effects.^40^, ^41^ It has shown preventive and therapeutic effects in experimental models of chronic inflammatory and autoimmune diseases.^42^, ^43^ However, the exact mechanism underlying the effects of Naringenin remains largely unknown. In our study, we explored the therapeutic potential of Naringenin in arthritis using CIA mice and assessed its potent pharmacological activity in suppressing macrophage‐mediated inflammatory responses. Although previous studies have proposed that Naringenin may ameliorate the CIA‐induced inflammatory response and synovial hyperplasia by inhibiting dendritic cell maturation,^37^ the mechanism elucidated in our study appears to involve the activation of the AMPK signaling pathway and the facilitation of macrophage autophagy. These findings contribute valuable insights towards the utilization of Naringenin as a natural therapeutic agent for RA.

RA is an autoimmune disease characterized by chronic joint inflammation and joint destruction.^44^ The occurrence of concomitant histopathological alterations can be linked to the activation and proliferation of inflammatory cells, the secretion of pro‐inflammatory cytokines, and the attrition of activated macrophages, which are the precursors of osteoclasts, within the cartilage. Macrophage activation leads to the release of a diverse array of inflammatory mediators, encompassing TNF‐α, IL‐1β, IL‐17, and TGF‐β. The complex interactions of inflammatory factors accompany the pathological process of RA.^45^ Studies have shown that blocking the inflammatory factor TNF‐α in RA significantly improves joint inflammation.^46^ As a pro‐inflammatory cytokine, IL‐1β plays a key role in the pathogenesis of arthritis.^47^ While the involvement of TGF‐β in inflammation remains a topic of debate, existing literature suggests that TGF‐β can stimulate the synthesis of pro‐inflammatory cytokines and chemokines, thereby intensifying the inflammatory cascade.^48^ Conversely, TGF‐β exhibits immunosuppressive properties by impeding the activation and proliferation of various immune cells, including T cells and natural killer cells.^49^ Moreover, TGF‐β has been found to facilitate the differentiation of regulatory T cells (Tregs).^50^ Nevertheless, it is noteworthy that individuals diagnosed with RA displayed substantially elevated levels of serum TGF‐β, specifically in those afflicted with arthritis.^51^ Suppression of TGF‐β proved effective in mitigating the bone resorption process in RA.^6^ In our investigation, we observed a significant decrease in the levels of TNF‐α, IL‐1β, and TGF‐β in the serum of CIA mice following Naringenin administration. This observation suggests that the potential inhibitory impact of Naringenin on RA in CIA mice could be attributed to the downregulation of inflammatory mediators.

Previous in vitro and in vivo studies have demonstrated that multiple pro‐inflammatory cytokines released by macrophage play an important role in the pathogenesis of various rheumatic diseases.^52^ Macrophage activation is dependent on oxidative stress injury and promotes the expression of pro‐inflammatory cytokines through activation of NF‐κB signaling pathway.^53^, ^54^ In this study, Naringenin enhanced antioxidant capacity in CIA mice, as clearly depicted in Figure 2. In addition, Naringenin reduced ROS levels in RAW264.7 cells. These results suggest that Naringenin may inhibit macrophage activation by regulating oxidative stress. In macrophages, autophagy is involved in maintaining redox equilibrium by removing cellular oxidative components. To investigate the underlying mechanism by which Naringenin enhances macrophage antioxidant capacity, we conducted a study on the impact of Naringenin on autophagy. Consistent with our expectations, treatment with Naringenin led to an upregulation of LC3A/B‐II expression and downregulation of p62 expression. Additionally, Naringenin activated the autophagy flux in RAW264.7 cells. Existing findings indicate that impaired autophagy may contribute to the development of RA, while promoting autophagy has shown to alleviate inflammatory symptoms associated with RA. Therefore, to confirm that Naringenin inhibits macrophage activation through initiation of autophagy, we blocked autophagy by knockdown of Atg5 or treatment of CQ. Figure 4 illustrates the reversal of macrophage activation inhibition caused by Naringenin when autophagy is suppressed. In recent years, activation of AMPK has been reported to regulate a variety of metabolic processes, including autophagy.^55^ AMPK directly promotes autophagy by phosphorylating the autophagy‐associated protein ULK1.^56^, ^57^ In this study, AMPK signaling was activated after Naringenin treatment, suggesting that Naringenin's effect on macrophage activation and autophagy may be related to AMPK activation. AMPK was further blocked by knockdown of Ampkα or treatment of Compound C, and Naringenin's inhibition of macrophage activation and initiation of autophagy was reversed. Therefore, activation of AMPK by Naringenin initiated autophagy and inhibited macrophage activation.

Although our initial findings indicate the ability of Naringenin to activate AMPK and induce autophagy while suppressing inflammation, further investigations are required to elucidate whether this mechanism remains relevant in an in vivo model of RA. Nevertheless, substantiating the mechanism of Naringenin in RA animal models poses considerable challenges due to the potential impact of autophagy inhibition on the inflammatory response.^58^ Furthermore, apart from macrophages, RA involves various other types of immune cells, all of which contribute significantly to the disease pathology.^59^ Therefore, it is imperative to conduct additional studies focusing on these immune cell populations to comprehensively address the research gap in our study.

In summary, the therapeutic effects of Naringenin on arthritis involve the reduction of joint inflammation and the induction of macrophage autophagy. Consequently, these two favorable outcomes of Naringenin collectively ameliorated symptoms in RA‐afflicted mice. The potential mechanism underlying Naringenin's actions could be attributed to the activation of AMPK. Thus, this investigation contributes additional substantiation for the potential utilization of Naringenin in the treatment of RA.

## AUTHOR CONTRIBUTIONS

**Wei Zhang**: Conceptualization; formal analysis; writing—original draft; writing—review & editing. **Yuan Zhang**: Writing—review & editing. **Jianguang Zhang**: Writing—review & editing. **Chunbiao Deng**: Writing—review & editing. **Chao Zhang**: Conceptualization; writing—original draft; writing—review & editing.

## CONFLICT OF INTERESTS STATEMENT

The authors declare no conflict of interest.

## ETHICS STATEMENT

The animal experimental protocol was reviewed and approved by the Experimental Animal Ethics Committee of North Sichuan Medical College (Nanchong, China).

## Data Availability

All data generated or analyzed during this study are included in this published article.

## References

[1] Nakada T , Mager DE . Systems model identifies baseline cytokine concentrations as potential predictors of rheumatoid arthritis inflammatory response to biologics. Br J Pharmacol. 2022;179:4063‐4077. 35355255 10.1111/bph.15845

[2] Tsujimoto S , Ozaki Y , Ito T , Nomura S . Usefulness of cytokine gene polymorphisms for the therapeutic choice in Japanese patients with rheumatoid arthritis. Int J Gen Med. 2021;14:131‐139. 33469350 10.2147/IJGM.S287505 PMC7813643

[3] Crispino N , Ciccia F . JAK/STAT pathway and nociceptive cytokine signalling in rheumatoid arthritis and psoriatic arthritis. Clin Exp Rheumatol. 2021;39:668‐675. 33200731

[4] Tweehuysen L , den Broeder AA , Schraa K , Netea MG , van den Hoogen FHJ , Joosten LAB . Predictive value of ex‐vivo drug‐inhibited cytokine production for clinical response to biologic DMARD therapy in rheumatoid arthritis. Clin Exp Rheumatol. 2019;37:367‐372. 30767874

[5] Monserrat Sanz J , Bohorquez C , Gomez AM , et al. Methrotexate treatment inmunomodulates abnormal cytokine expression by T CD4 lymphocytes present in DMARD‐Naive rheumatoid arthritis patients. Int J Mol Sci. 2020;21:6847. 32961930 10.3390/ijms21186847 PMC7555887

[6] Hernandez G , Mills TS , Rabe JL , et al. Pro‐inflammatory cytokine blockade attenuates myeloid expansion in a murine model of rheumatoid arthritis. Haematologica. 2020;105:585‐597. 31101752 10.3324/haematol.2018.197210 PMC7049366

[7] Kondo N , Kuroda T , Kobayashi D . Cytokine networks in the pathogenesis of rheumatoid arthritis. Int J Mol Sci. 2021;22:10922. 34681582 10.3390/ijms222010922 PMC8539723

[8] Kataoka T , Naoe S , Murakami K , et al. Mechanisms of action of radon therapy on cytokine levels in normal mice and rheumatoid arthritis mouse model. J Clin Biochem Nutr. 2022;70:154‐159. 35400822 10.3164/jcbn.21-91 PMC8921724

[9] Sun HT , Li JP , Qian WQ , Yin MF , Yin H , Huang GC . Quercetin suppresses inflammatory cytokine production in rheumatoid arthritis fibroblast‐like synoviocytes. Exp Ther Med. 2021;22:1260. 34603528 10.3892/etm.2021.10695 PMC8453329

[10] Maloley PM , England BR , Sayles H , et al. Post‐traumatic stress disorder and serum cytokine and chemokine concentrations in patients with rheumatoid arthritis(). Semin Arthritis Rheum. 2019;49:229‐235. 30777365 10.1016/j.semarthrit.2019.02.002 PMC6687567

[11] Du H , Zhang X , Zeng Y , et al. A novel phytochemical, DIM, inhibits proliferation, migration, invasion and TNF‐α induced inflammatory cytokine production of synovial fibroblasts from rheumatoid arthritis patients by targeting MAPK and AKT/mTOR signal pathway. Front Immunol. 2019;10:1620. 31396207 10.3389/fimmu.2019.01620 PMC6663984

[12] Raza K . Early rheumatoid arthritis is characterised by a distinct and transient synovial fluid cytokine profile of T cell and stromal cell origin. Arthritis Res Ther. 2019;21:226. 31694702 10.1186/s13075-019-2026-4 PMC6836325

[13] Zhao C , Li XY , Li ZY , Li M , Liu ZD . Moxibustion regulates T‐regulatory/T‐helper 17 cell balance by modulating the microRNA‐221/suppressor of cytokine signaling 3 axis in a mouse model of rheumatoid arthritis. J Integr Med. 2022;25(3):453‐462. 10.1016/j.joim.2022.06.002 35729047

[14] Samarpita S , Rasool M . Cyanidin attenuates IL‐17A cytokine signaling mediated monocyte migration and differentiation into mature osteoclasts in rheumatoid arthritis. Cytokine. 2021;142:155502. 33810944 10.1016/j.cyto.2021.155502

[15] Giwa R , Brestoff JR . Mitochondria transfer to CD4(+) T cells may alleviate rheumatoid arthritis by suppressing pro‐inflammatory cytokine production. Immunometabolism. 2022;4(2):e220009. 35444834 10.20900/immunometab20220009 PMC9017867

[16] Mahmood Z , Schmalzing M , Dörner T , Tony HP , Muhammad K . Therapeutic cytokine inhibition modulates activation and homing receptors of peripheral memory B cell subsets in rheumatoid arthritis patients. Front Immunol. 2020;11:572475. 33042152 10.3389/fimmu.2020.572475 PMC7518039

[17] Qasim S , Alamgeer P , Kalsoom S , et al. Rosuvastatin attenuates rheumatoid arthritis‐associated manifestations via modulation of the pro‐ and anti‐inflammatory cytokine network: a combination of in vitro and in vivo studies. ACS Omega. 2021;6:2074‐2084. 33521447 10.1021/acsomega.0c05054 PMC7841959

[18] Boutet MA , Courties G , Nerviani A , et al. Novel insights into macrophage diversity in rheumatoid arthritis synovium. Autoimmun Rev. 2021;20:102758. 33476818 10.1016/j.autrev.2021.102758

[19] Bruijnen STG , Verweij NJF , Gent YYJ , et al. Imaging disease activity of rheumatoid arthritis by macrophage targeting using second generation translocator protein positron emission tomography tracers. PLoS One. 2019;14:e0222844. 31553762 10.1371/journal.pone.0222844 PMC6760780

[20] Cao Y , Liu J , Huang C , et al. Wilforlide A ameliorates the progression of rheumatoid arthritis by inhibiting M1 macrophage polarization. J Pharmacol Sci. 2022;148:116‐124. 34924115 10.1016/j.jphs.2021.10.005

[21] Cutolo M , Campitiello R , Gotelli E , Soldano S . The role of M1/M2 macrophage polarization in rheumatoid arthritis synovitis. Front Immunol. 2022;13:867260. 35663975 10.3389/fimmu.2022.867260 PMC9161083

[22] Demarco B , Danielli S , Fischer FA , Bezbradica JS . How pyroptosis contributes to inflammation and fibroblast‐macrophage cross‐talk in rheumatoid arthritis. Cells. 2022;11:1307. 35455985 10.3390/cells11081307 PMC9028325

[23] Xu A , Yang R , Zhang M , et al. Macrophage targeted triptolide micelles capable of cGAS‐STING pathway inhibition for rheumatoid arthritis treatment. J Drug Target. 2022;30(9):961‐972. 35467469 10.1080/1061186X.2022.2070173

[24] Han X , Kou J , Zheng Y , et al. ROS generated by upconversion Nanoparticle‐mediated photodynamic therapy induces autophagy via PI3K/AKT/mTOR signaling pathway in M1 peritoneal macrophage. Cell Physiol Biochem. 2019;52:1325‐1338. 31050281 10.33594/000000093

[25] Ilyas G , Cingolani F , Zhao E , Tanaka K , Czaja MJ . Decreased macrophage autophagy promotes liver injury and inflammation from alcohol. Alcohol: Clin Exp Res. 2019;43:1403‐1413. 30964198 10.1111/acer.14041 PMC6602853

[26] Li XY , Wang YJ , Chen S , et al. Laminaria japonica polysaccharide suppresses atherosclerosis via regulating autophagy‐mediated macrophage polarization. J Agric Food Chem. 2022;70:3633‐3643. 35167294 10.1021/acs.jafc.1c07483

[27] Li Z , Fu WJ , Chen XQ , et al. Autophagy‐based unconventional secretion of HMGB1 in glioblastoma promotes chemosensitivity to temozolomide through macrophage M1‐like polarization. J Exp Clin Cancer Res. 2022;41:74. 35193644 10.1186/s13046-022-02291-8 PMC8862393

[28] Liang J , Liu J , Tang Y , et al. Sophoridine inhibits endotoxin‐induced acute lung injury by enhancing autophagy of macrophage and reducing inflammation. J Leukoc Biol. 2022;112:115‐125. 35603481 10.1002/JLB.3MA0322-428R

[29] Taşlıdere A , Türkmen NB , Ciftci O , Aydın M . Investigation into the protective effects of Naringenin in phthalates‐induced reproductive damage. Eur Rev Med Pharmacol Sci. 2022;26:3419‐3429. 35647821 10.26355/eurrev_202205_28835

[30] Zhang X , Huang Y , Shi Y , et al. Naringenin ultrafine powder was prepared by a new anti‐Solvent recrystallization method. Nanomaterials. 2022;12:12 2108. 10.3390/nano12122108 PMC9231401 35745448

[31] Liu JF , Chang TM , Chen PH , et al. Naringenin induces endoplasmic reticulum stress‐mediated cell apoptosis and autophagy in human oral squamous cell carcinoma cells. J Food Biochem. 2022;46(11):e14221. 35596593 10.1111/jfbc.14221

[32] Motallebi M , Bhia M , Rajani HF , et al. Naringenin: A potential flavonoid phytochemical for cancer therapy. Life Sci. 2022;305:120752. 35779626 10.1016/j.lfs.2022.120752

[33] Rauf A , Shariati MA , Imran M , et al. Comprehensive review on naringenin and naringin polyphenols as a potent anticancer agent. Environ Sci Pollut Res. 2022;29:31025‐31041. 10.1007/s11356-022-18754-6 35119637

[34] Liu Z , Niu X , Wang J . Naringenin as a natural immunomodulator against T cell‐mediated autoimmune diseases: literature review and network‐based pharmacology study. Crit Rev Food Sci Nutr. 2022;7:1‐18. 10.1080/10408398.2022.2092054 35776085

[35] Hajizadeh A , Abtahi Froushani SM , Tehrani AA , Azizi S , Hashemi B . Effects of naringenin on experimentally induced rheumatoid arthritis in wistar rats. Arch Razi Inst. 2021;76:903‐912. 35096326 10.22092/ari.2020.351612.1527 PMC8790977

[36] Aihaiti Y , Song Cai Y , Tuerhong X , et al. Therapeutic effects of naringin in rheumatoid arthritis: network pharmacology and experimental validation. Front Pharmacol. 2021;12:672054. 34054546 10.3389/fphar.2021.672054 PMC8160516

[37] Li YR , Chen DY , Chu CL , et al. Naringenin inhibits dendritic cell maturation and has therapeutic effects in a murine model of collagen‐induced arthritis. J Nutr Biochem. 2015;26:1467‐1478. 26350255 10.1016/j.jnutbio.2015.07.016

[38] Brand DD , Latham KA , Rosloniec EF . Collagen‐induced arthritis. Nat Protoc. 2007;2:1269‐1275. 17546023 10.1038/nprot.2007.173

[39] Inglis JJ , Šimelyte E , McCann FE , Criado G , Williams RO . Protocol for the induction of arthritis in C57BL/6 mice. Nat Protoc. 2008;3:612‐618. 18388943 10.1038/nprot.2008.19

[40] Yousuf M , Shamsi A , Khan S , et al. Naringenin as a potential inhibitor of human cyclin‐dependent kinase 6: molecular and structural insights into anti‐cancer therapeutics. Int J Biol Macromol. 2022;213:944‐954. 35690164 10.1016/j.ijbiomac.2022.06.013

[41] Yadav B , Vishwakarma V , Kumar S , Aggarwal NK , Gupta R , Yadav A . Ameliorative role of naringenin against lead‐induced genetic damage and oxidative stress in cultured human lymphocytes. J Biochem Mol Toxicol. 2022;36:e23036. 35289026 10.1002/jbt.23036

[42] Jasemi SV , Khazaei H , Fakhri S , Mohammadi‐Noori E , Farzaei MH . Naringenin improves ovalbumin‐Induced allergic asthma in rats through antioxidant and anti‐inflammatory effects. Evid‐Based Compl Alterna Med. 2022;2022:1‐10. 10.1155/2022/9110798 PMC9001106 35419072

[43] Sanson C , Boukaiba R , Houtmann S , et al. The grapefruit polyphenol naringenin inhibits multiple cardiac ion channels. Naunyn‐Schmiedeberg's Arch Pharmacol. 2022;395:735‐740. 35412073 10.1007/s00210-022-02240-4

[44] Patschan S , Bothmann L , Patschan D , et al. Association of cytokine patterns and clinical/laboratory parameters, medication and periodontal burden in patients with rheumatoid arthritis (RA). Odontology. 2020;108:441‐449. 32300908 10.1007/s10266-020-00517-9 PMC7250790

[45] Peng S , Hu C , Liu X , et al. Rhoifolin regulates oxidative stress and proinflammatory cytokine levels in Freund's adjuvant‐induced rheumatoid arthritis via inhibition of NF‐κB. Braz J Med Biol Res. 2020;53:e9489. 32401927 10.1590/1414-431X20209489 PMC7233197

[46] Qasim S , Alamgeer P , Saleem M , et al. Appraisal of the antiarthritic potential of prazosin via inhibition of proinflammatory cytokine TNF‐α: a key player in rheumatoid arthritis. ACS Omega. 2021;6:2379‐2388. 33521476 10.1021/acsomega.0c05698 PMC7841939

[47] Ptacek J , Hawtin RE , Sun D , et al. Diminished cytokine‐induced Jak/STAT signaling is associated with rheumatoid arthritis and disease activity. PLoS One. 2021;16:e0244187. 33444321 10.1371/journal.pone.0244187 PMC7808603

[48] Liu F , Qiu H , Xue M , et al. MSC‐secreted TGF‐β regulates lipopolysaccharide‐stimulated macrophage M2‐like polarization via the Akt/FoxO1 pathway. Stem Cell Res Ther. 2019;10:345. 31771622 10.1186/s13287-019-1447-y PMC6878630

[49] Shaim H , Shanley M , Basar R , et al. Targeting the alphav integrin/TGF‐beta axis improves natural killer cell function against glioblastoma stem cells. J Clin Invest. 2021;131(14):e142116. 34138753 10.1172/JCI142116 PMC8279586

[50] Korn T , Bettelli E , Oukka M , Kuchroo VK . IL‐17 and Th17 cells. Annu Rev Immunol. 2009;27:485‐517. 19132915 10.1146/annurev.immunol.021908.132710

[51] Charlier E , Deroyer C , Ciregia F , et al. Chondrocyte dedifferentiation and osteoarthritis (OA). Biochem Pharmacol. 2019;165:49‐65. 30853397 10.1016/j.bcp.2019.02.036

[52] Hannemann N , Apparailly F , Courties G . New insights into macrophage heterogeneity in rheumatoid arthritis. Joint Bone Spine. 2021;88:105091. 33130232 10.1016/j.jbspin.2020.105091

[53] Steinz MM , Ezdoglian A , Khodadust F , et al. Folate receptor beta for macrophage imaging in rheumatoid arthritis. Front Immunol. 2022;13:819163. 35185910 10.3389/fimmu.2022.819163 PMC8849105

[54] Zhou X , Huang D , Wang R , et al. Targeted therapy of rheumatoid arthritis via macrophage repolarization. Drug Delivery. 2021;28:2447‐2459. 34766540 10.1080/10717544.2021.2000679 PMC8592611

[55] Wang Q , Chen J , Zhang M , et al. Autophagy induced by muscarinic acetylcholine receptor 1 mediates migration and invasion targeting Atg5 via AMPK/mTOR pathway in prostate cancer. J Oncol. 2022;2022:6523195. 35720225 10.1155/2022/6523195 PMC9203210

[56] Fu Z , Wu X , Zheng F , Zhang Y . Activation of the AMPK‐ULK1 pathway mediated protective autophagy by sevoflurane anesthesia restrains LPS‐induced acute lung injury (ALI). Int Immunopharmacol. 2022;108:108869. 35605434 10.1016/j.intimp.2022.108869

[57] Balaha MF , Almalki ZS , Alahmari AK , Ahmed NJ , Balaha MF . AMPK/mTOR‐driven autophagy & Nrf2/HO‐1 cascade modulation by amentoflavone ameliorates indomethacin‐induced gastric ulcer. Biomed Pharmacother. 2022;151:113200. 35676791 10.1016/j.biopha.2022.113200

[58] Ahmedy OA , Salem HH , Sayed NH , Ibrahim SM . Naringenin affords protection against lipopolysaccharide/d‐galactosamine‐induced acute liver failure: role of autophagy. Arch Biochem Biophys. 2022;717:109121. 35065059 10.1016/j.abb.2022.109121

[59] Jiang Q , Yang G , Liu Q , Wang S , Cui D . Function and role of regulatory T cells in rheumatoid arthritis. Front Immunol. 2021;12:626193. 33868244 10.3389/fimmu.2021.626193 PMC8047316

